# Clinical and pathological indicators for predicting disease-free survival after radical surgery in elderly patients with early-stage triple-negative invasive ductal carcinoma of the breast

**DOI:** 10.3389/fonc.2026.1922118

**Published:** 2026-08-14

**Authors:** Tao Zhang, Yingli Guo, Xue Li, Lingling Xie

**Affiliations:** 1 Department of Biotherapy, Cancer Center and State Key Laboratory of Biotherapy, West China Hospital, Sichuan University, Chengdu, China; 2 State Key Laboratory of Biotherapy and Cancer Center, West China Hospital, Sichuan University, Chengdu, China; 3 Sichuan Provincial Center for Gynaecology and Breast Diseases, The Affiliated Hospital of Southwest Medical University, Luzhou, Sichuan, China

**Keywords:** disease-free survival (DFS), elderly, invasive ductal carcinoma (IDC), nomogram, recurrence prediction, triple-negative breast cancer (TNBC)

## Abstract

**Background:**

Elderly patients with early-stage triple-negative breast cancer are underrepresented in clinical trials, and recurrence prediction models tailored to this population are lacking. We aimed to develop a prediction tool using routinely available clinicopathological indicators.

**Methods:**

We retrospectively enrolled 215 women aged ≥60 years with stage IA–IIB triple-negative invasive ductal carcinoma who underwent radical or modified radical mastectomy. Candidate predictors were screened by univariate Cox regression and entered into a multivariable Cox model with backward elimination. A nomogram was constructed and internally validated using bootstrap resampling. Discrimination was assessed by Harrell’s C-index and time-dependent area under the curve (AUC). Risk groups were defined using tertiles of the model-derived risk score. Decision curve analysis compared the net benefit with TNM stage alone.

**Results:**

Four variables were retained: TNM stage, Ki-67 percentage, neutrophil-to-lymphocyte ratio (NLR), and postoperative chemotherapy. The bootstrap-corrected C-index was 0.793. Time-dependent AUCs were 0.934, 0.866, and 0.796 at 1, 3, and 5 years, respectively. The three risk groups showed clearly separated disease-free survival curves. The nomogram yielded a higher net benefit than TNM stage alone across threshold probabilities of 5% to 40%.

**Conclusions:**

All model inputs were derived from standard preoperative evaluations and postoperative treatment records. For an older population with uneven access to molecular biomarkers, this nomogram can inform adjuvant treatment decisions using data already available. External validation in an independent cohort is needed before clinical adoption.

## Introduction

Triple-negative breast cancer (TNBC) is defined by the absence of estrogen receptor, progesterone receptor, and HER2 expression (1). The disease represents 10% to 20% of all breast cancers, but its survival rate is the lowest among all subtypes, lower than that of hormone receptor-positive and HER2-positive disease (2, 3). Because endocrine therapy and anti-HER2 agents have no target in TNBC, chemotherapy remains the backbone of systemic treatment, though its efficacy is far from satisfactory (4).

TNBC encompasses several histological types. Significant histological heterogeneity exists within TNBC, which complicates prognostic modeling. We therefore restricted our cohort to invasive ductal carcinoma (IDC), the most prevalent subtype, accounting for more than 80% of cases (5, 6). Metaplastic carcinoma responds poorly to standard chemotherapy (7). Medullary carcinoma, despite its aggressive microscopic appearance, often follows a more indolent course (8). Adenoid cystic carcinoma rarely metastasizes (9, 10). These distinct histological subtypes exhibit divergent clinical behaviors and responses to standard chemotherapy, necessitating different management strategies. A model built on a mix of these types would have to capture biological variation that has little to do with the predictors we set out to test. We avoided that problem by focusing on IDC. BRCA1/2 mutations are present in approximately 15% to 20% of patients with TNBC, and these tumors respond better to platinum agents and PARP inhibitors (11). However, the role of BRCA1/2 mutations in predicting postsurgical recurrence in this population remains incompletely defined. PD-L1, expressed in 20% to 40% of tumors, guides immunotherapy decisions but adds little to postoperative risk stratification (12, 13). A pragmatic challenge is the limited accessibility of these biomarkers outside centers with genetic testing platforms or standardized immunohistochemistry.

The connection between systemic inflammation, nutritional status, and solid tumor outcomes has been supported by a decade of data (14, 15). The neutrophil-to-lymphocyte ratio (NLR) and platelet-to-lymphocyte ratio (PLR) condense peripheral blood counts into measures of systemic inflammation (16, 17). The prognostic nutritional index (PNI) combines serum albumin with the lymphocyte count to capture nutritional and immune status in a single measure. Studies of colorectal, gastric, and lung cancers have validated the prognostic value of these indices (18–20).

Among breast cancer patients, inflammatory markers measured five years after diagnosis tracked late recurrence. The same pattern has been observed in the neoadjuvant therapy setting, where lower inflammatory indices distinguished responders from non-responders. In advanced TNBC, a high inflammatory state is associated with worse outcomes (21–24). This evidence comes largely from advanced disease or the neoadjuvant setting. Whether NLR, PLR, and PNI can provide prognostic information independent of anatomical staging for postoperative recurrence prediction in early-stage TNBC has been investigated only to a limited extent (25, 26). These studies have provided a foundation for risk stratification in TNBC. They also highlight important gaps: none targeted patients aged ≥60 years with stage IA–IIB IDC-type TNBC, and the majority included mixed histological subtypes or wide age ranges, weakening the signal for older patients.

Older patients represent a critical knowledge gap. Patients with TNBC aged ≥60 years are frequently excluded from clinical trials because of comorbidities and functional status, or are given reduced treatment intensity (27–29). A biomarker cutoff set for younger patients may not translate to older populations. No recurrence prediction model has been specifically developed for this population.

We retrospectively enrolled a cohort of female patients with TNBC aged ≥60 years with stage IA to IIB disease and histologically confirmed IDC. All patients underwent radical or modified radical mastectomy. We used routinely available preoperative clinical and pathological indicators to construct a model predicting disease-free survival (DFS). This model provides individualized recurrence estimates for a population that is underrepresented in clinical trials. It can inform adjuvant treatment decisions and serve as a baseline tool for stratifying older patients with TNBC as targeted-immunotherapy combinations become more accessible.

## Methods

### Patient selection

We screened female patients with TNBC who underwent radical or modified radical mastectomy at West China Hospital, Sichuan University, from May 2019 to May 2022 in this single-center retrospective study. Inclusion criteria were as follows: age ≥60 years; stage IA to IIB; histological type of invasive ductal carcinoma; immunohistochemically confirmed TNBC; and no neoadjuvant therapy before surgery. Exclusion criteria were a history of other malignant tumors, follow-up shorter than six months, and missing clinical data exceeding 30%. The last follow-up date was 30 March 2026.

### Data collection

We extracted the following variables from the electronic medical record system. Demographic and host characteristics included age, body mass index (BMI), menopausal status, smoking history, alcohol history, history of cardiovascular disease, and history of diabetes. Pathological characteristics included TNM stage, WHO histological grade, Ki-67 index, and CK5/6 status. Clinical parameters (measured within one month before surgery) included routine blood parameters, blood glucose, serum albumin, CA15-3, and carcinoembryonic antigen (CEA). Ki-67 percentage was entered as a continuous variable, with the hazard ratio reported per 10% increase.

Postoperative treatment information included chemotherapy and immunotherapy. From the preoperative complete blood count and albumin, we derived three indices: NLR = neutrophil count/lymphocyte count, PLR = platelet count/lymphocyte count, and PNI = serum albumin (g/L) + 5 × lymphocyte count (×10^9^/L).

Disease-free survival (DFS) was measured from the date of surgery. The first occurrence of locoregional recurrence, distant metastasis, or death from any cause was considered an event. Patients without an event at the last follow-up were censored.

From the collected variables, we prespecified a set of candidate predictors for univariate screening. Neutrophil count, lymphocyte count, and albumin were not tested individually because they are components of NLR, PLR, and PNI. Entering both raw components and derived indices would create redundancy. We tested only the composite indices.

### Model building and evaluation

Candidate variables were screened using univariate Cox regression. A threshold of P < 0.1 was chosen to retain potentially important predictors that might show modest univariate associations but gain significance after adjustment. Those meeting this criterion were entered into a multivariable Cox proportional-hazards model. We then applied backward elimination with P < 0.05.

We assessed discrimination with Harrell’s C-index. The apparent C-index was calculated from the original sample. We then generated 200 bootstrap resamples and computed a corrected C-index from the average optimism. Time-dependent receiver operating characteristic (ROC) curves were generated using the cumulative/dynamic definition of sensitivity and specificity. Based on the optimal cutoff values derived from the time-dependent ROC curves at 36 and 60 months (by maximizing the Youden index), we calculated sensitivity, specificity, positive predictive value (PPV), and negative predictive value (NPV). We constructed a nomogram for predicting DFS probabilities based on the final Cox model. Calibration curves at 36 and 60 months were generated using bootstrap resampling. Calibration slopes and calibration-in-the-large were extracted from the bootstrap calibration procedure. The heuristic shrinkage factor was calculated as (model χ² – df)/model χ².

### Risk stratification and decision curve analysis

We assigned each patient a linear predictor from the final Cox model and divided the cohort into groups at the tertiles of its distribution. We labeled these groups low risk, medium risk, and high risk. Differences in DFS among the three groups were displayed using Kaplan–Meier curves, and the log-rank test was used to compare survival distributions.

Decision curve analysis was performed to compare the net benefit of the final model with that of the TNM stage. Net benefit quantifies the clinical value of a model by subtracting the weighted harm of false positives from the benefit of true positives at a given threshold probability. The weight reflects the odds of the threshold. The threshold probability ranged from 0% to 40%.

### Statistical software

All analyses were performed in R version 4.6.0. Group comparisons were made using the Student’s t-test, Wilcoxon rank-sum test, chi-square test, or Fisher’s exact test, according to variable type and distribution. Cox proportional-hazards models were used for univariate and multivariable survival analyses. Two-sided P < 0.05 was considered statistically significant.

## Results

### Baseline characteristics of the cohort

We enrolled 215 patients. The patient selection process is summarized in **Supplementary Figure 1**.

During follow-up, 58 patients (27.0%) developed a DFS event. Age, BMI, menopausal status, smoking history, alcohol history, and comorbidities were comparable between the recurrence and non-recurrence groups (**Table 1**). Patients who recurred had more advanced TNM stage: T1N0M0 accounted for 17.2% of the recurrence group versus 40.8% of the non-recurrence group, while T2N1M0 was present in 37.9% versus 12.1%. WHO grade III tumors predominated in the recurrence group (91.4% vs. 71.3%). The Ki-67 percentage was higher among patients who recurred (mean, 56% vs. 34%). NLR and PLR were higher in the recurrence group, and PNI was lower. Postoperative chemotherapy was less frequent in the recurrence group (41.4% vs. 59.2%). Immunotherapy rates were low in both groups and did not differ.

**Table 1:** Baseline characteristics of elderly patients with early-stage triple-negative invasive ductal carcinoma of the breast.

Characteristic	Non-recurrence group (n = 157)	Recurrence group (n = 58)	*P*
Age (y), mean ± SD	66.31 ± 4.94	66.16 ± 6.40	0.263
BMI, mean ± SD	22.60 ± 2.73	23.03 ± 3.34	0.372
Menopausal status			0.998
Yes	153 (97.5%)	56 (96.6%)	
No	4 (2.5%)	2 (3.4%)	
Menopausal age	50.07 ± 5.25	49.34 ± 4.36	0.467
Alcohol history			0.735
Yes	15 (9.6%)	4 (6.9%)	
No	142 (90.4%)	54 (93.1%)	
Smoking history			0.397
Yes	19 (12.1%)	4 (6.9%)	
No	138 (87.9%)	54 (93.1%)	
Cardiovascular disease			0.189
Yes	61 (38.9%)	29 (50.0%)	
No	96 (61.1%)	29 (50.0%)	
Diabetes			0.258
Yes	61 (38.9%)	17 (29.3%)	
No	96 (61.1%)	41 (70.7%)	
TNM Stage			<0.001
T1N0M0	64 (40.8%)	10 (17.2%)	
T1N1M0	17 (10.8%)	7 (12.1%)	
T2N0M0	55 (35.0%)	19 (32.8%)	
T2N1M0	19 (12.1%)	22 (37.9%)	
T3N0M0	2 (1.3%)	0 (0.0%)	
Ki-67 status			0.037
Positive	122 (77.7%)	53 (91.4%)	
Negative	35 (22.3%)	5 (8.6%)	
Ki-67 percentage (%)	34 ± 23	56 ± 23	<0.001
CK5/6			0.729
Positive	108 (68.8%)	42 (72.4%)	
Negative	49 (31.2%)	16 (27.6%)	
WHO grade			0.004
I/II	45 (28.7%)	5 (8.6%)	
III	112 (71.3%)	53 (91.4%)	
CEA (ng/mL), mean ± SD	3.99 ± 2.79	3.99 ± 3.38	0.641
NLR, mean ± SD	3.56 ± 1.72	4.37 ± 2.26	<0.001
PLR, mean ± SD	146.48 ± 44.48	167.68 ± 59.93	0.005
PNI, mean ± SD	48.21 ± 3.48	46.76 ± 4.52	0.003
Plasma glucose (mmol/L), mean ± SD	6.92 ± 1.96	6.71 ± 1.67	0.872
RBC (×10^12^/L), mean ± SD	4.22 ± 0.44	4.08 ± 0.49	0.051
Hemoglobin (g/L), mean ± SD	117.53 ± 9.12	116.78 ± 9.39	0.943
WBC (×10^9^/L), mean ± SD	7.73 ± 1.08	7.98 ± 1.62	0.906
Neutrophil (×10^9^/L), mean ± SD	4.94 ± 0.88	5.28 ± 1.24	0.044
Lymphocyte (×10^9^/L), mean ± SD	1.51 ± 0.41	1.37 ± 0.5	0.003
Albumin (g/L), mean ± SD	40.69 ± 2.94	39.92 ± 3.66	0.116
Postoperative chemotherapy			0.029
Yes	93 (59.2%)	24 (41.4%)	
No	64 (40.8%)	34 (58.6%)	
Postoperative immunotherapy			0.397
Yes	19 (12.1%)	4 (6.9%)	
No	138 (87.9%)	54 (93.1%)	

BMI, body mass index; CEA, carcinoembryonic antigen; NLR, neutrophil-to-lymphocyte ratio; PLR, platelet-to-lymphocyte ratio; PNI, prognostic nutritional index; RBC, red blood cell; WBC, white blood cell.

### Model development

A total of 22 variables were screened by univariate Cox regression (**Table 2**). Of those, 8 met the P < 0.1 threshold: TNM stage IIA and IIB versus IA, Ki-67 percentage, WHO grade III, NLR, PLR, PNI, RBC, and postoperative chemotherapy status. Age, BMI, menopausal status, smoking history, alcohol history, cardiovascular disease, diabetes, CK5/6, CEA, blood glucose, and postoperative immunotherapy status all had P ≥ 0.1 and were excluded.

**Table 2:** Univariate Cox regression analyses of candidate variables for DFS in elderly patients with early-stage triple-negative invasive ductal carcinoma.

Variable	Hazard ratio	95% confidence interval	*P*
Age (y), mean ± SD	1	0.951–1.051	0.994
BMI, mean ± SD	1.056	0.965–1.156	0.235
Menopausal status			
Yes	0.651	0.159–2.669	0.551
No	ref	ref	/
Menopausal age	0.972	0.924–1.023	0.277
Alcohol history			
Yes	0.731	0.265–2.02	0.548
No	ref	ref	/
Smoking history			
Yes	0.599	0.217–1.656	0.324
No	ref	ref	/
Cardiovascular disease			
Yes	1.430	0.851–2.405	0.177
No	ref	ref	/
Diabetes			
Yes	0.747	0.423–1.317	0.313
No	ref	ref	/
TNM Stage			
Stage IA	ref	ref	/
Stage IIA	2.410	1.129–5.143	0.023
Stage IIB	6.477	2.977-14.092	<0.001
Ki-67 percentage (per 10% increase)	1.35	1.22–1.49	<0.001
CK5/6			
Positive	1.339	0.742–2.415	0.332
Negative	ref	ref	/
WHO Grade			
I/II	ref	ref	/
III	4.775	1.728–13.196	0.003
CEA	0.999	0.913–1.093	0.981
NLR	1.161	1.068–1.262	0.001
PLR	1.008	1.003–1.012	0.001
PNI	0.909	0.857–0.963	0.001
Plasma glucose	0.965	0.833–1.116	0.629
RBC	0.539	0.326–0.893	0.016
Hemoglobin	0.989	0.963–1.015	0.388
Postoperative chemotherapy			
Yes	0.517	0.304–0.877	0.015
No	ref	ref	/
Postoperative immunotherapy			
Yes	0.568	0.205–1.569	0.275
No	ref	ref	/

Ki-67 percentage was entered as a continuous variable (range 0–1). The hazard ratio is reported per 10% increase. BMI, body mass index; CEA, carcinoembryonic antigen; NLR, neutrophil-to-lymphocyte ratio; PLR, platelet-to-lymphocyte ratio; PNI, prognostic nutritional index; RBC, red blood cell.

We fitted a full multivariable model with all eight candidates. After backward elimination, four variables were retained (**Table 3**): TNM stage IIB (HR 8.74, 95% CI 3.85–19.82, P < 0.001), Ki-67 percentage (HR 1.31 per 10% increase, 95% CI 1.16–1.47, P < 0.001), NLR (HR 1.15, 95% CI 1.02–1.28, P = 0.018), and postoperative chemotherapy (HR 0.45, 95% CI 0.26–0.81, P = 0.007). Stage IIA was kept alongside IIB to preserve the stage ordering (HR 2.09, 95% CI 0.97–4.48, P = 0.049). WHO grade, PLR, PNI, and RBC were eliminated.

**Table 3:** Multivariable Cox regression analysis of DFS in elderly patients with early-stage triple-negative invasive ductal carcinoma.

Variable	Hazard ratio	95% confidence interval	*P*
TNM stage			
Stage IA	ref	ref	/
Stage IIA	2.086	0.971–4.482	0.049
Stage IIB	8.735	3.851–19.816	<0.001
Ki-67 percentage (per 10% increase)	1.31	1.16–1.47	<0.001
WHO grade			
I/II	ref	ref	/
III	1.369	0.452–4.145	0.578
RBC	0.932	0.445–1.953	0.852
NLR	1.146	1.024–1.282	0.018
PLR	0.999	0.994–1.005	0.856
PNI	0.944	0.876–1.018	0.136
Postoperative chemotherapy			
Yes	0.454	0.256–0.806	0.007
No	ref	ref	/

The Ki-67 percentage was entered as a continuous variable (range 0–1, representing 0%–100% positivity). The hazard ratio is reported per 10% increase. RBC, red blood cell; NLR, neutrophil-to-lymphocyte ratio; PLR, platelet-to-lymphocyte ratio; PNI, prognostic nutritional index.

**Figure 1:**
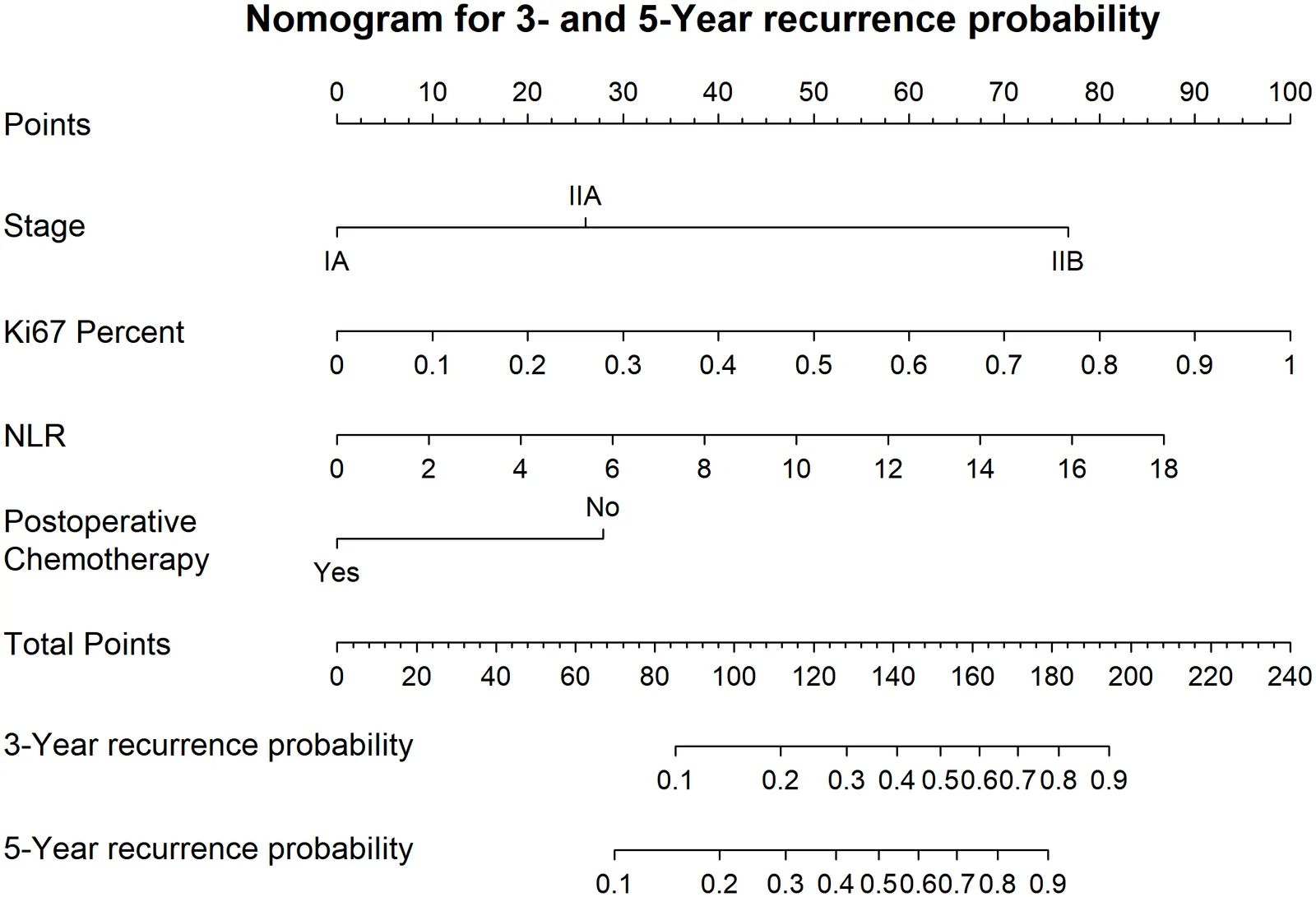
Nomogram for predicting 3-year and 5-year DFS in elderly patients with early-stage triple-negative invasive ductal carcinoma. The nomogram estimates the probability of disease-free survival at 3 and 5 years after radical surgery. To use it, locate each predictor on its axis, draw a vertical line to the points scale, and sum the points. The total points correspond to the predicted recurrence probability on the bottom scales. Postoperative chemotherapy: no = 0, yes = 1.

A nomogram was constructed from these predictors to estimate 3-year and 5-year DFS probabilities (**Figure 1**). TNM stage IIB and Ki-67 percentage carried the largest weights in the point scale. To illustrate, we selected a patient from the cohort: stage IIA, Ki-67 of 95%, NLR of 3.31, and postoperative chemotherapy received. Her predicted 3-year recurrence probability was 36.6%, and the 5-year probability was 50.5%.

### Discrimination and calibration

**Figure 2:**
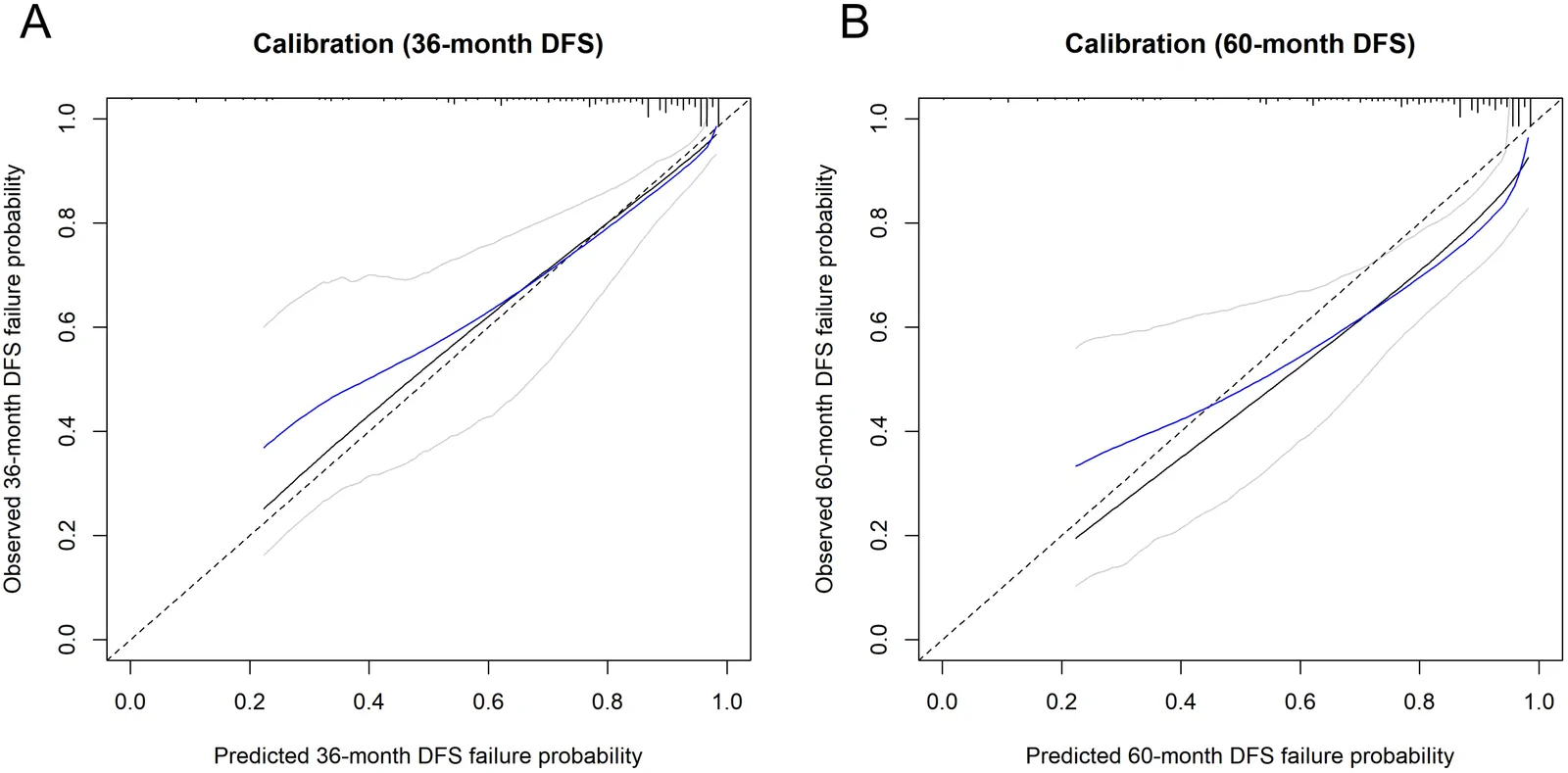
Calibration curves of the nomogram for predicting 3-year **(A)** and 5-year **(B)** DFS. Calibration curves compare predicted probabilities from the nomogram with observed probabilities. The diagonal dashed line represents perfect agreement. Bootstrap resampling was used for internal validation. The 36-month **(A)** and 60-month **(B)** both show close agreement between predicted and observed values across most of the risk spectrum, with slight deviation in the upper probability range at 60 months.

**Figure 2** shows the bootstrap calibration curves. At 36 months, the calibration slope was 0.747, and the intercept was 0.198; predicted and observed probabilities agreed reasonably well. At 60 months, the slope was 0.720, and the intercept was 0.127. The curve fell slightly below the diagonal when predicted risk exceeded roughly 60%, indicating that the nomogram overestimated risk in that upper range. The apparent C-index was 0.804. After correcting for optimism with 200 bootstrap resamples, it settled at 0.793. The heuristic shrinkage factor was 0.933, indicating modest overfitting.

**Figure 3:**
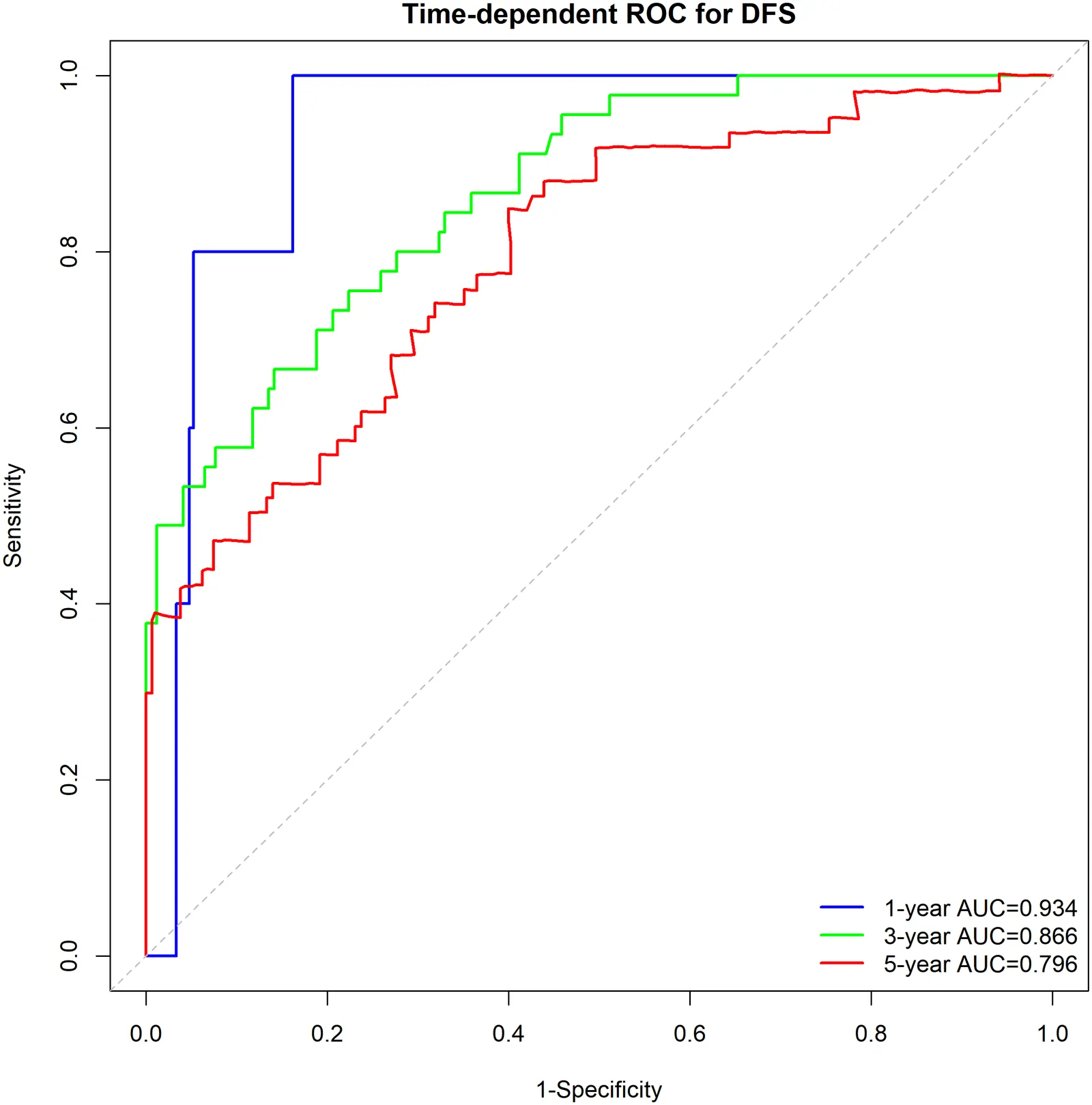
Time-dependent receiver operating characteristic curves for predicting disease-free survival at 1, 3, and 5 years. The curves display the discriminatory ability of the nomogram at three follow-up landmarks. The blue line corresponds to 1 year, the green line to 3 years, and the red line to 5 years. The area under the curve (AUC) for each time point is reported in the figure.

The time-dependent ROC curves appear in **Figure 3**. The AUC was 0.934 at 1 year, 0.866 at 3 years, and 0.796 at 5 years. **Table 4** presents the classification performance at 36 and 60 months. At 36 months, accuracy reached 0.772, sensitivity was 0.756, specificity was 0.777, PPV was 0.472, and NPV was 0.923. At 60 months, sensitivity increased to 0.860, while specificity decreased to 0.595; PPV and NPV were 0.434 and 0.922, respectively.

**Table 4:** Discrimination performance of the nomogram for DFS at 36 and 60 months.

Time of model	AUC	Accuracy	Sensitivity	Specificity	PPV	NPV	C-index	C-index (corrected)
36 months	0.866	0.772	0.756	0.777	0.472	0.923	0.804	0.793
60 months	0.796	0.665	0.860	0.595	0.434	0.922	0.804	0.793

### Risk stratification and decision curve analysis

**Figure 4:**
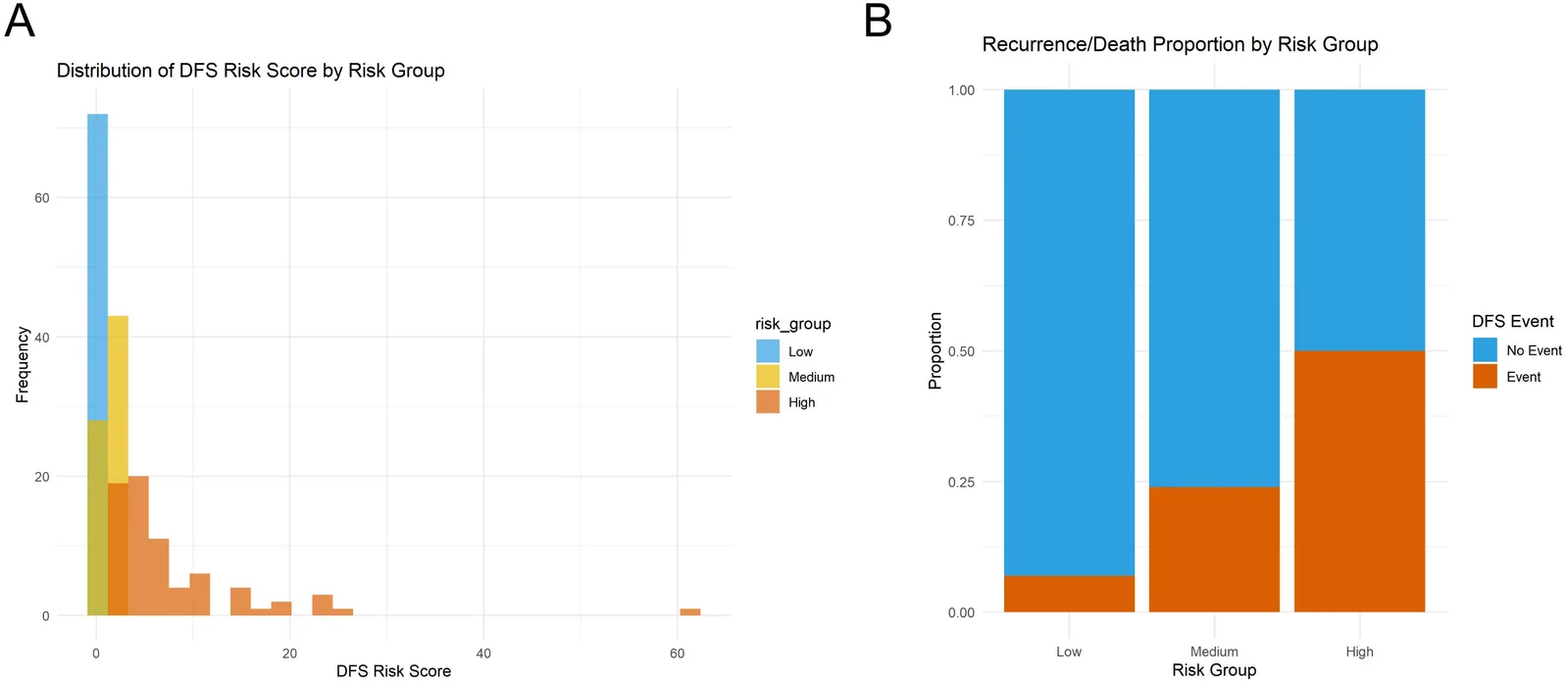
Distribution of disease-free survival risk scores and event proportions across risk groups. **(A)** shows the density of risk scores in the low-, medium-, and high-risk groups. **(B)** displays the percentage of patients who experienced a DFS event (recurrence) versus those who remained event-free within each risk stratum.

We computed a risk score for each patient from the final Cox model and divided the cohort into three groups by tertiles of the score. **Figure 4A** shows the distribution of scores within each stratum. The low‑risk group clustered tightly near zero, with a peak density around 72. The medium‑risk group spanned scores from roughly 0 to 4, with a lower peak density around 43. The high‑risk group showed a wider spread, concentrated between 0 and 15, with a small tail extending toward 25 and an isolated outlier near 60. Events were unevenly distributed across groups (**Figure 4B**). In the low‑risk group, 6% of patients had a DFS event versus 94% who remained event‑free. The medium‑risk group showed a 24% event proportion. In the high‑risk group, half of the patients experienced an event and half did not.

**Figure 5:**
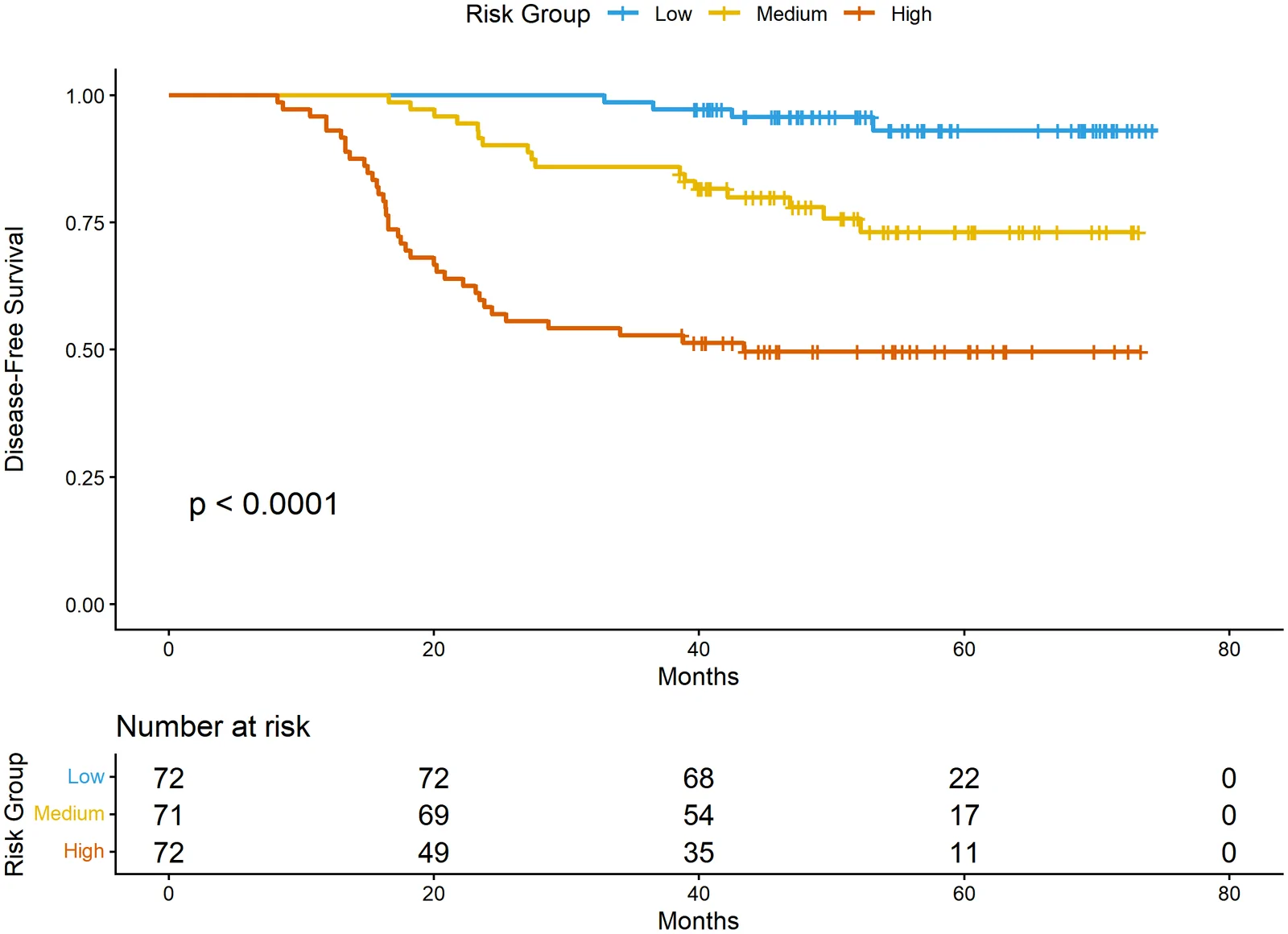
Kaplan–Meier curves of DFS stratified by risk group. DFS curves for the low-, medium-, and high-risk groups defined by tertiles of the nomogram-derived risk score. The number of patients at risk at each time point is shown below the x-axis. The log-rank test was used to compare survival distributions across the three groups.

Kaplan–Meier curves for the three risk groups are shown in **Figure 5**. The curves separated early, with the high-risk group diverging within the first 12 months (log-rank P < 0.0001).Three-year DFS was 98.6% (low risk), 85.9% (medium risk), and 52.8% (high risk). At five years, the rates fell to 93.0%, 73.1%, and 49.6%, respectively. At 24 months, the DFS rate in the high-risk group had already decreased to 58.3%, compared with 90.1% in the medium-risk group and 100% in the low-risk group.

**Figure 6:**
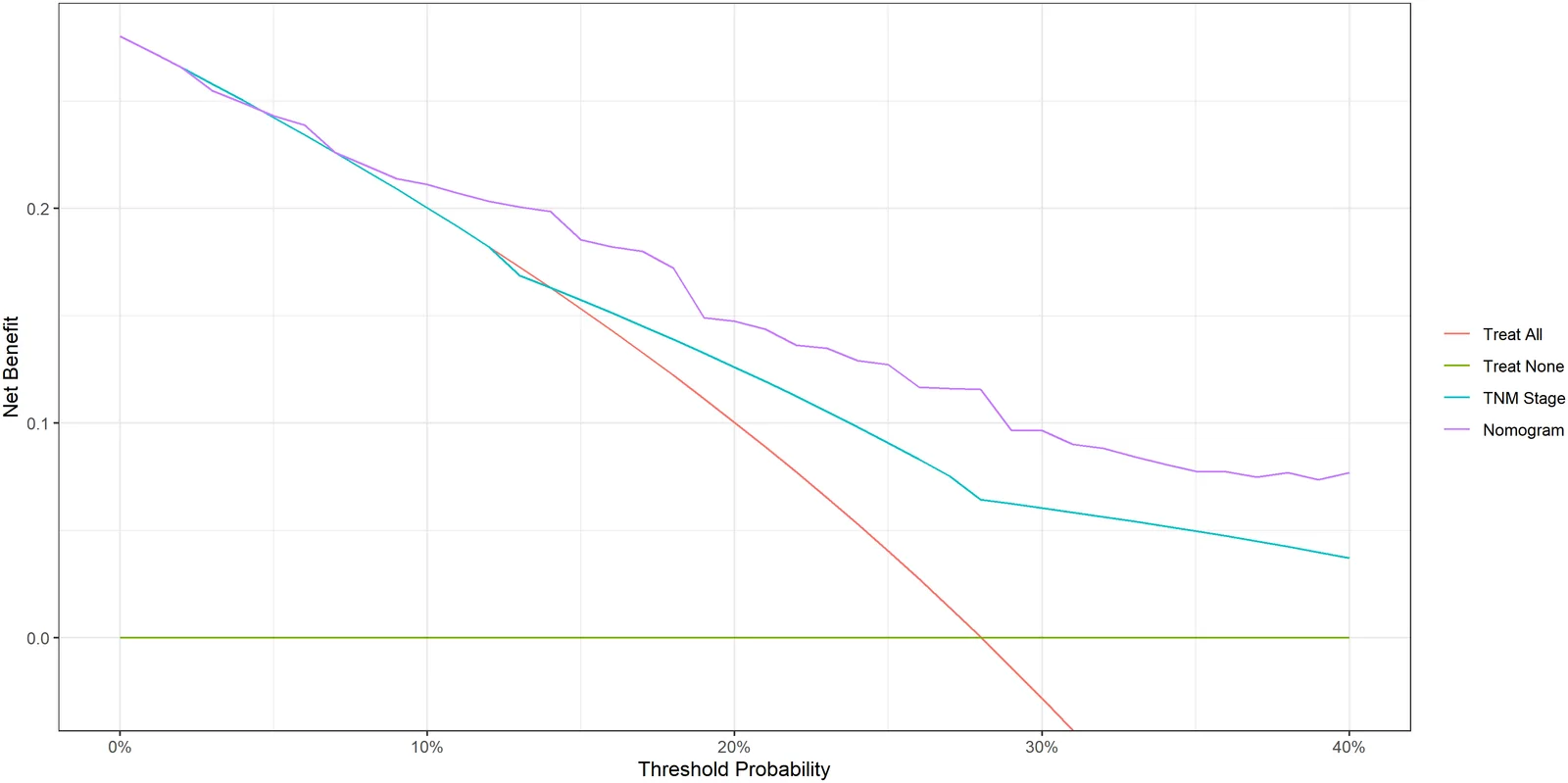
Decision curve analysis comparing the net benefit of the nomogram and TNM stage for predicting DFS. The y-axis represents net benefit, and the x-axis represents threshold probability. The purple line corresponds to the nomogram, the cyan line to TNM stage alone, the red line to treating all patients, and the green line to treating none. The nomogram yielded a higher net benefit than TNM stage across threshold probabilities of roughly 5% to 40% at both time points.

Decision curve analysis compared the nomogram with TNM stage alone (**Figure 6**). The nomogram yielded a higher net benefit than TNM stage across threshold probabilities of 5% to 40%. At the threshold of 20%, the net benefit of the nomogram was approximately 0.15 compared with 0.12 for TNM stage. At 30%, the nomogram still showed a net benefit of approximately 0.10, while TNM stage had dropped to roughly 0.06. The nomogram curve remained above the TNM stage curve across the clinically relevant range of threshold probabilities. It outperformed the “treat all” strategy once the threshold probability exceeded 10%.

## Discussion

We built a recurrence prediction model for elderly patients with early-stage triple-negative invasive ductal carcinoma. Four routinely available indicators were retained after backward elimination: TNM stage, Ki-67 percentage, NLR, and postoperative chemotherapy. Risk group stratification yielded three distinct survival curves. Across a threshold probability range of 5% to 40%, the nomogram yielded a higher net benefit than TNM stage alone.

The four variables in the final model are routinely available in clinical practice. TNM stage IIB carried the heaviest weight in the nomogram, which is expected given that the anatomical extent of disease is the backbone of postoperative risk assessment in early breast cancer. Ki-67 percentage, modeled as a continuous variable per 10% increase, contributed the second largest weight in the nomogram. High Ki-67 is consistently linked to recurrence in TNBC, and its contribution in our older cohort reinforces its value as a proliferation marker (30, 31). NLR remained independently significant after adjustment for stage and Ki-67. NLR reflects the systemic inflammatory milieu, which in older patients may capture both tumor-driven inflammation and age-related immune alterations (32). Previous studies reported the prognostic value of NLR in TNBC, but they largely included younger patients. Our finding extends this evidence to an exclusively older population (33–35).

Postoperative chemotherapy was associated with a reduced hazard of recurrence. Patients who received chemotherapy likely differed from those who did not in ways we could not fully capture, including performance status, physician judgment, and patient preference. We retained chemotherapy because it improved model fit and reflects the real-world treatment context, but this association should not be interpreted causally. We recorded chemotherapy as a binary variable. The interval from surgery to chemotherapy initiation, specific regimens, and treatment completion were not captured. Immortal time bias cannot be entirely ruled out because patients who started chemotherapy must have survived long enough to receive it. In our practice, however, adjuvant chemotherapy for early TNBC is generally started within 4 to 8 weeks of surgery, a window during which recurrence is uncommon in stage IA-IIB disease. The majority of patients in the no-chemotherapy group were not intended to receive chemotherapy from the outset. The HR of 0.45 should therefore be interpreted as an association reflecting both treatment effect and patient selection rather than as a causal estimate. It may modestly overstate the true protective effect.

The inclusion of postoperative chemotherapy means that the nomogram is designed for postoperative use, after the decision to administer chemotherapy has been made and treatment has been completed. It is not intended to guide the initial chemotherapy decision. Rather, it identifies patients who remain at high risk despite having completed standard treatment and who may benefit from intensified surveillance or, when available, additional adjuvant therapy. A version of the model without the chemotherapy variable could be explored for preoperative risk stratification in future studies.

We acknowledge that variable selection using univariate screening followed by backward elimination can yield optimistic estimates when the number of events is modest relative to the number of candidates. Our univariate analysis involved 22 candidate variables for 58 events, giving an events-per-variable ratio of approximately 2.6 at the screening stage, which is well below the conventional threshold of 10. The P < 0.1 screening threshold was a pragmatic choice: a stricter cutoff risks excluding variables that gain significance only after adjustment, while a more liberal one would inflate the number of candidates and worsen overfitting. Previous studies have also adopted this approach (36–38). LASSO or other penalized regression methods can produce more stable variable selection in small samples and were considered. We opted for backward elimination because our priority was a clinically interpretable model with a small number of variables and because the bootstrap-corrected C-index showed only modest optimism. The calibration slopes at 36 and 60 months were 0.747 and 0.720, respectively, with positive intercepts, reflecting slight overestimation at high predicted risk. The heuristic shrinkage factor was 0.933, suggesting that overfitting was limited. Applying this factor would shrink the nomogram coefficients toward zero and could further improve calibration in external data. External validation with penalized or model-averaged approaches would help establish whether the same four predictors consistently emerge in independent data.

Earlier prognostic models for TNBC have combined clinical, pathological, and hematological parameters. They provided a foundation for risk stratification (39–42). They also left gaps: none targeted patients aged ≥60 years with stage IA–IIB IDC-type TNBC, and the majority included mixed histological subtypes or wide age ranges, weakening the signal for older patients. By restricting our cohort to IDC and to patients aged 60 years and above, we reduced two sources of heterogeneity that have limited earlier models.

An accurate prediction tool does more than estimate risk; it informs adjuvant treatment decisions. The adjuvant treatment landscape for early TNBC has shifted with KEYNOTE-522, which established pembrolizumab in the neoadjuvant and adjuvant setting for high-risk disease, and OlympiA, which demonstrated the benefit of adjuvant olaparib in germline BRCA-mutated HER2-negative breast cancer (43, 44). These advances depend on biomarker-based selection: PD-L1 for pembrolizumab and BRCA status for olaparib (45). Nevertheless, in our cohort, only 23 patients (10.7%) received adjuvant immunotherapy, mostly pembrolizumab. No patient received a PARP inhibitor. BRCA testing was not systematically performed, and PARP inhibitors were not approved or reimbursed for adjuvant early TNBC in China during the enrollment period. This highlights a substantial disparity between the evidence from pivotal clinical trials and the therapeutic reality for this older patient population in our setting. Our model was therefore designed to utilize readily available clinical and pathological parameters. A model built from preoperative blood tests and standard pathology can identify high-risk patients regardless of whether molecular testing or novel agents are accessible (46–48). When these therapies become accessible, the same tool could guide de-escalation therapy. Our nomogram requires no assays beyond standard preoperative workup.

From a clinical perspective, the three risk groups suggest distinct management approaches. Patients in the low-risk group could be considered for standard or reduced-intensity surveillance. Those in the medium-risk group should continue routine adjuvant treatment and follow-up. The high-risk group may warrant intensified surveillance, including more frequent imaging during the first 2–3 years, and should be prioritized for clinical trials evaluating novel adjuvant strategies in TNBC. These recommendations are illustrative, and formal studies evaluating the impact of risk-stratified follow-up on outcomes are needed before clinical implementation.

Our study has some limitations. The nomogram was developed exclusively in patients with IDC, the dominant histological subtype of TNBC. Its performance in metaplastic, medullary, adenoid cystic, and other special histological subtypes is unknown. Given the distinct biological behavior and clinical management of these subtypes, the nomogram should not be applied outside IDC until validated in those populations. Treatment variables were captured as binary indicators, and detailed timing and regimen data were unavailable. Follow-up was relatively short for early-stage disease. Late recurrences occurring beyond the follow-up window would not have been captured, and the 5-year estimates may shift as more events accumulate. In an older population, non-cancer deaths may act as competing risks. The number of deaths unrelated to breast cancer was not separately adjudicated. In cohorts with longer follow-up, a competing risks framework would yield more accurate estimates of the cumulative incidence of recurrence. Risk groups were defined by tertiles of the risk score derived from our own data. These cutoffs are sample-dependent and will shift when the nomogram is applied to new populations. Until fixed thresholds are established through external validation, the continuous risk score should be used for individual risk estimation, and the group assignments reported here should be viewed as illustrative.

The nomogram was internally validated by bootstrapping. It has not been tested in an independent cohort, and external validation in an independent cohort is mandatory before clinical application. A prospective multicenter cohort that includes patients receiving contemporary immunotherapy would test whether these findings hold and whether the model can guide treatment de-escalation in low-risk groups.

## Conclusion

We developed a recurrence prediction model for elderly patients with early-stage triple-negative invasive ductal carcinoma using four routinely available indicators: TNM stage, Ki-67 percentage, NLR, and postoperative chemotherapy. The nomogram stratified patients into distinct prognostic groups and outperformed TNM stage alone in clinical decision-making across a broad range of threshold probabilities.

For an older population that is underrepresented in clinical trials and for whom access to molecular biomarker testing and novel therapeutics remains uneven, a tool of this kind can inform adjuvant treatment decisions and follow-up intensity using data already available. It may also provide a baseline for treatment de-escalation as targeted and immunotherapeutic options become more broadly available. External validation in an independent multicenter cohort is required before clinical adoption.

## Data Availability

The raw data supporting the conclusions of this article will be made available by the authors without undue reservation.

## Supplementary material

Supplementary Figure 1 Patient selection flowchart.
